# Effectiveness of seasonal malaria chemoprevention in reducing under-five malaria morbidity and mortality in the Savannah Region, Ghana

**DOI:** 10.4314/gmj.v56i2.2

**Published:** 2022-06

**Authors:** Michael R Adjei, Chrysantus Kubio, Marcel Buamah, Adjei Sarfo, Thomas Suuri, Saeed Ibrahim, Abubakari Sadiq, Ihsan I Abubakari, Janet V Baafi

**Affiliations:** 1 Regional Health Directorate, Ghana Health Service, Damongo, Savannah Region, Ghana; 2 National Malaria Control Programme, Tamale, Northern Region, Ghana; 3 Sunyani West District Health Directorate, Ghana Health Service, Fiapre, Bono Region, Ghana

**Keywords:** Seasonal malaria chemoprevention, Savannah Region, malaria, SiCapp, DHIMS-2

## Abstract

**Objective:**

To assess the effectiveness of seasonal malaria chemoprevention (SMC) in reducing under-five malaria morbidity and mortality

**Design:**

Under-five malaria data for confirmed episodes, deaths, and number of children dosed per cycle of SMC campaign were extracted from the District Health Information Management System (DHIMS-2) for 2018–2019. Data verification was done to compare extracted data with the source for completeness and consistency. Association between SMC and the main outcome variables (malaria cases and mortality) was computed from 2X2 tables and reported as rate ratios at a 95% confidence level.

**Setting:**

All seven (7) districts in Savannah Region, Ghana

**Participants:**

Children under five years

**Intervention:**

Sulphadoxine-Pyrimethamine and Amodiaquine (SPAQ) prophylaxis given monthly, four times, durng the rainy season (July to October)

**Main outcome measures:**

SMC coverage per cycle and under-five malaria morbidity and mortality ratios

**Results:**

Over 370,000 dose packs of SPAQ were administered with an average cycle coverage of 93%. There was approximately 17% (p<0.01) and 67% (p=0.047) reduction in malaria-related morbidity and mortality, respectively, in the implementation year compared with the baseline. This translated into nearly 9,300 episodes of all forms of malaria and nine malaria-attributable deaths averted by the intervention.

**Conclusion:**

SMC (combined with existing control measures) wields prospects of accelerating the regional/national malaria elimination efforts if the implementation is optimised. Expansion of the intervention to other high-prevalence regions with seasonal variation in disease burden may be worthwhile.

**Funding:**

None declared

## Introduction

Malaria is a life-threatening disease caused by parasites transmitted to humans through the bites of infected female Anopheles mosquitoes. In 2018, an estimated 228 million malaria cases were recorded globally, with over 400,000 deaths. Children under five years are the most vulnerable and account for 67% of the mortalities. Ninety-four per cent (94%) of cases and 93% of deaths were from Africa.^1^

Malaria accounts for approximately 40% of outpatient attendance in Ghana, with children and pregnant women being the most vulnerable.^2^

**Figure 1:**
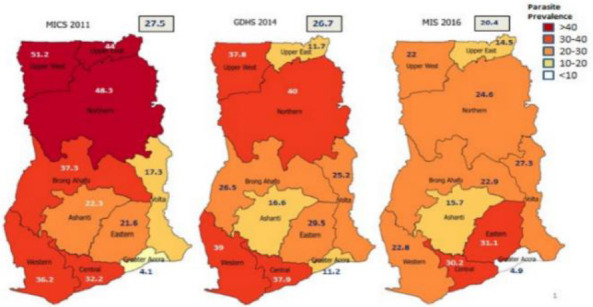
Malaria parasite prevalence among children under five years of age by region, Ghana; 2011, 2014, & 2016

Like most West African countries, Ghana is classified to be in the control phase according to the global malaria elimination programme.^3^ The country's malaria control interventions pre-dates its independence having switched from monotherapy to artemisinin-based combination therapies (ACTs) in 2004; deployment of insecticide-treated bed nets (ITNs) in 2004; and limited introduction of indoor residual spraying (IRS) in 2005.^4^ These interventions and many others (case management, intermit-tent preventive treatment for malaria in pregnancy, etcetera) have significantly contributed to the malaria control progress (Figures 1). The malaria case fatality rate has decreased from 0.52 in 2015 to 0.12 in 2018.^5^

Achieving further control and setting the tone for elimination requires additional evidence-based interventions targeting the parasite in the host (human beings) or the vector (anopheles mosquito). Across the Sahel, most malaria illnesses and deaths occur during the rainy season, typically between July and October. Seasonal Malaria Chemoprevention (SMC) is a highly effective intervention to prevent malaria during this peak transmission period among children under five – the most at risk. The intervention, as recommended by World Health Organization (WHO), is safe, cost-effective, and feasible and can prevent up to 75 % of malaria cases in under-fives when used alongside other malaria interventions.^6^

In 2015, the National Malaria Control Programme successfully piloted SMC in the Upper West Region of Ghana. The intervention contributed to approximately 42% reduction in malaria parasite prevalence in 2016.^5^ Outcome of the study necessitated expansion to include Upper East Region and Northern Region (now divided into three – Northern, North East, and Savannah Regions) in 2016 and 2019 respectively.^7^ However, no evaluation of the intervention had been conducted in the Savannah Region following its implementation. The study aimed to assess the effectiveness of SMC in reducing under-five malaria morbidity and mortality. The findings will contribute to programme strengthening and further research, particularly within the region.

## Methods

### Study setting

Savannah Region is one of the 16 regions in Ghana and was carved out of the Northern Region in 2019. It has the largest land surface area (35,862 km^2^), and the estimated populations for 2018 and 2019 were 513,687 and 525,803, respectively. Children 3–59 months and those under five years constitute approximately 19.2% and 20% respectively.^8^

**Figure 2:**
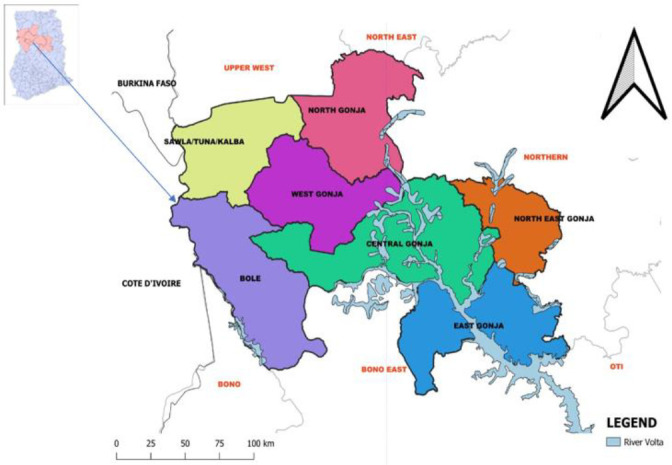
Map of Savannah Region; 2020. Source: Savannah Regional Health Directorate

There are seven (7) administrative districts (Figure 2) and a total of 1,063 communities. Health facilities include three (3) District hospitals, two (2) polyclinics; 23 health centres; 108 community-based health planning and services (CHPS) compounds; and nine (9) private clinics. Health care financing is mainly through the National Health Insurance Scheme (NHIS). OPD attendance rate for July-December 2018 was 0.93 and 0.90 for the same period in 2019, an indication of appreciable health care service utilisation in the region.^8^ Malaria constitutes approximately 34.5% of OPD attendance.^9^

Health-seeking behaviour varies by gender and age. Women (especially those who are pregnant) and caregivers seeking care for their children (under five years) often patronise NHIS-accredited health facilities. However, there is poor health-seeking behaviour among males (especially adults): the majority resort to traditional medicine and only report to health facilities in the event of failure.^8^

**Figure 3:**
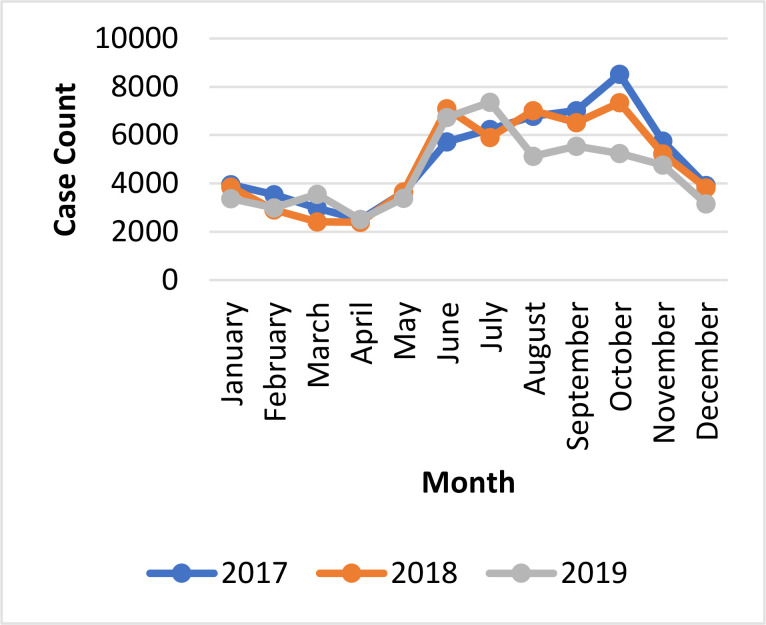
Seasonal trend of under-five malaria cases, Savannah Region, Ghana; 2017-2019 (Source: DHIMS2)

Agriculture is the major source of livelihood, with a considerable proportion being peasant farmers. Housing is mainly constructed with mud and roofed thatch, especially in rural areas. Most communities (>50%) are located more than 5 km from the nearest health facility.^8^ There is generally a poor road network, and access is worsened during the rainy season (July to October) due to springing up of water bodies and overflow of rivers.. The rainy season coincides with the peak transmission period of malaria (Figure 3) as a result of increased proliferation of the vector (female anopheles mosquito).

### Implementation of SMC

**Figure 4:**
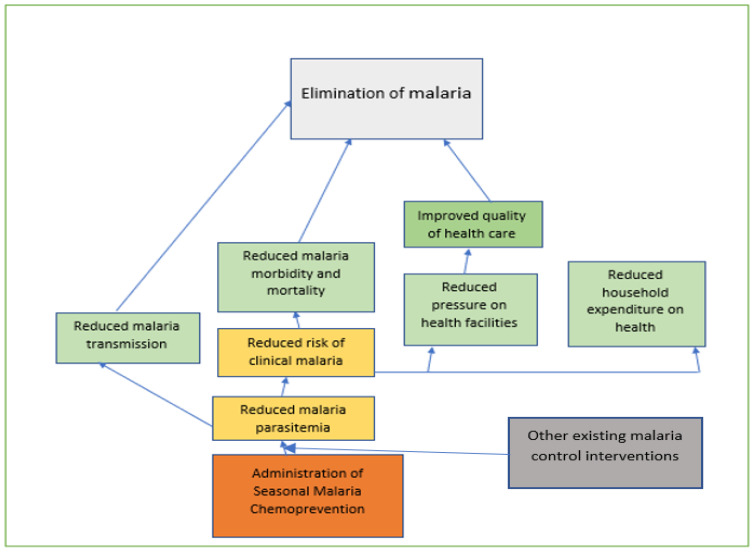
Conceptual framework for malaria elimination (Source: Authors')

SMC involves administering four monthly courses of two antimalarial drugs, sulphadoxine-pyrimethamine (SP) and amodiaquine (AQ), to children 3–59 months, and it is delivered through a household-to-household approach. The objective of the intervention is to achieve and maintain therapeutic blood concentration of the antimalaria to reduce parasitemia^10^ and, consequently, malaria episodes (Figure 4).

Community-based Surveillance Volunteers (CBSVs) and supervisors are trained before deployment to the field. CBSVs visit households, register children in the target group onto a mobile application called SMC in Children app (SiCapp), and administer the intervention (SPAQ).

SiCapp is an android application developed by the National Malaria Control Programme to replace the paper-based campaign data management system. It has a volunteer interphase that enables registration, dosing, and logistics accountability as well as a supervisor interphase that facilitates in-process monitoring. Data collected on SiCapp include age of child, dose of SPAQ administered, and adverse drug reaction (ADR) among others. Field data is synchronised by volunteers and supervisors onto a central server for collation and analysis.

Eligible children fall into two broad categories: 3–11 months and 12–59 months. Sick children and those with known allergies to SPAQ are excluded. A child is assessed and the appropriate dosage of anti-malaria is handed to a caregiver who administers the first dose (SPAQ) in the presence of the volunteer. The child is observed for a minimum of five minutes for ADR. The remaining two tablets of AQ are given to the caregiver to administer on the second and third days respectively. The volunteer follows up to observe and document drug administration as well as refer children with ADR to the nearest clinic for treatment at no cost to the caregivers.

Each volunteer doses 20–40 children per day and dosing details are entered onto SiCapp. To ensure quality campaigns, monitoring and supervision are carried out at the community, sub-district, district and regional levels. Supervisors visit volunteers on the field to provide technical support as needed. Caregivers are also interviewed to evaluate the performance of volunteers as well as the acceptability of the intervention. Volunteers-supervisor meetings are held at the end of each day to account for logistics, resolve challenges, and sync data to a central server.

Review meetings are organised at the end of each cycle to disseminate performance feedback and sharing of vital lessons. The SiCapp data is validated and entered onto the SMC summary return forms in District Health Information Management System (DHIMS-2) – a national health data platform.

### Study design

Analysis of routine data was conducted on under-five malaria data entered onto DHIMS-2 for 2018 and 2019 respectively.

### Data collection

Under-five malaria data for 2018–2019 was extracted from DHIMS-2. DHIMS-2 is free open-source software (https://www.dhis2.org) developed from the district health information system (DHIS). It was first introduced in Ghana in the year 2007 and was primarily used for collecting and reporting health data. Data is collected at the health facility level using standard registers, collated onto summary forms and entered on the DHIMS-2 platform monthly or quarterly. The interphase is password protected and accessible to healthcare managers and health partners.

Data from all health facilities reporting on the DHIMS-2 platform were included in the study. The pre-intervention and intervention data were collected for the period 2018 and 2019 respectively. Under-five malaria data for July to December were extracted from outpatient (OPD) morbidity forms, malaria score card, in-patient records and SMC summary return forms onto Microsoft Excel 14.0 spreadsheet for analysis. Although SMC is implemented from July to October, data for November and December were included due to possible “spill-over” effects of the intervention beyond October.

Variables collected include the number of confirmed malaria episodes (uncomplicated or severe malaria diagnosed after 28 days of the previous infection) malaria deaths; and the number of children dosed per cycle of SMC. Data verification was carried out to compare extracted data with the source for completeness and consistency.

### Ethical consideration

Ethical approval was sought from Kintampo Health Research Centre Institutional Ethics Committee (KHR-CIEC); study approval number: KHRCIEC/2021-02. Administrative permission was sought from the Regional Director of Health Service for Savannah Region and the respective Municipal/District Directors of Health Service. Informed consent was sought from the caregivers of the participants. Confidentiality and anonymity were ensured throughout the process. Soft and hard copies of data were kept on a password-protected computer and under lock and key respectively.

### Data analysis

Extracted data was exported into Epi Info statistical soft-ware (Epi Info version 7.2.2.16, www.cdc.gov>epiinfo) for analysis. The proportion of children dosed with SPAQ per cycle was calculated by dividing the number dosed by the population of children 3–59 months (projected populations from Ghana Statistical Service) and multiplied by 100% (Figure 3). The number of malaria episodes per 1000 population was calculated by dividing the total number of confirmed episodes for each period by the population of children under five and multiplied by 1000 (Table 2). Malaria mortality per 1,000 was calculated by a similar approach (Table 2).

**Table 2:** Estimated rate ratio for under-five malaria morbidity and mortality during SMC implementation, Savannah Region; 2018–2019

Outcome	Rate (/1000/year)			Rate Ratio (95% CI)	p-value	Standardized	p-value
	Pre-implementation	Implementation				Rate Ratio	
		Observed	Expected			(95% CI)	
**All malaria**	512	410	423	0.80	0.000	0.83	0.000
				(0.79–0.81)		(0.82–0.83)	
**Uncomplicated malaria**	491	391	404	0.80	0.000	0.82	0.000
				(0.79–0.8)		(0.81–0.83)	
**Severe malaria**	21	18	19	0.85	0.000	0.90	0.000
				(0.8–0.91)		(0.83–0.94)	
**Malaria mortality**	0.12	0.04	0.04	0.33	0.047	0.33	0.047
				(0.11–1)		(0.11–1)	

Association between SMC and the main outcome variables (malaria cases and mortality) was computed from 2X2 tables and reported as a rate ratio at 95% confidence level.^11^ The effects of variations in health-seeking behaviour and data reporting (between the periods) on the outcome variables were controlled by indirect standardisation using the outpatient department (OPD) attendance rate of the pre-implementation period (2018) to calculate the expected number of cases and mortalities for the implementation period. The standardised rate ratios were then computed (Table 2). The effectiveness of the intervention was assessed by estimating the number of malaria cases and deaths averted in the implementation period (2019) compared with the baseline (2018).

## Results

**Figure 5:**
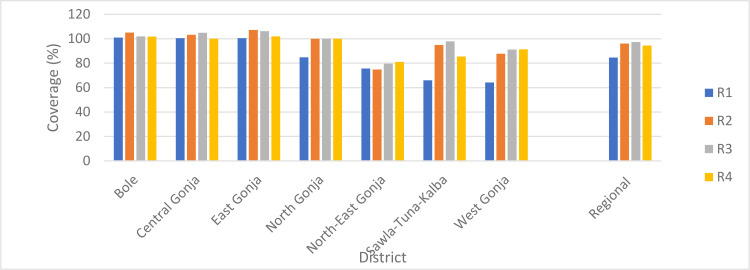
Coverage of SMC per cycle by district, Savannah Region; 2019

More than 95,000 episodes of malaria and 16 related deaths among children under five years were recorded during the study period (Table 1). The majority (95%) of the episodes were uncomplicated malaria and nearly 82% occurred among children 12–59 months. Approximately 55% and 75% of the episodes and deaths respectively, were recorded in the pre-implementation period (2018). Over 370,000 dose packs of SPAQ were administered to eligible children in the four-cycle campaign carried out in the intervention period (2019). Approximately 85% were administered to children 12–59 months. Generally, coverage increased sequentially from cycle one (C1) through cycle three (C3) and decreased in cycle four (C4) (Figure 5).

**Table 1:** Distribution of under-five malaria cases and deaths by district, Savannah Region; 2018–2019

District	2018		2019	
	Number of Malaria Cases	Number of Malaria Deaths	Number of Malaria Cases	Number of Malaria Deaths
**Bole**	16,457	3	16,426	1
**Central Gonja**	8,758	1	6,846	0
**East Gonja**	3,995	2	2,799	1
**North Gonja**	2,660	0	1,742	0
**North-East Gonja**	841	0	561	0
**Sawla-Tuna-Kalba**	13,081	5	11,455	2
**West Gonja**	6,836	1	3,238	0
**Savannah Region**	52,628	12	43,067	4

On average, 93% of children (3–59 months) received the intervention per cycle and the average number of treatments per child was 3.7 dose packs.

There was approximately 17% (RR=0.83; 95%CI: 0.82–0.83; p<0.01) and 67% (RR=0.33; 95% CI: 0.11–1; p=0.047) reduction in malaria-related morbidity and mortality respectively in the implementation period (2019) compared with the baseline (2018) (Table 2). This translated into nearly 9,300 episodes of all forms of malaria and nine deaths averted by the intervention. Approximately 97% of the averted episodes were uncomplicated malaria. The average completeness and consistency between extracted and source data were 91.3% and 89.5% respectively.

## Discussion

The observed reduction in under-five malaria morbidity and mortality can largely be attributed to the implementation of SMC because it was the only additional malaria control tool introduced in the region in 2019. In addition, the regional health system remained stable regarding leadership and staff turnover; and there were no changes in the implementation of other malaria control interventions during the study period.

The OPD attendance rate depends on health service utilisation and documentation of the same by health facilities. Variations could be real or apparent. The association between SMC and the outcome variables (malaria morbidity and mortality) could therefore be confounded by variations in health-seeking behaviour and data entry anomalies between the periods. However, this was mitigated through indirect standardisation: the number of cases and deaths that would have occurred in the implementation period (if it had the OPD attendance rate of the pre-implementation period) was calculated before computing the respective rate ratios (Table 2). Standardisation made the rates comparable and justified attribution of the observed difference to the intervention.

The reduction in the episodes of malaria during the intervention year is in tandem with the findings of other stud-ies.^12^,^13^,^14^ However, while our study observed a 17% drop in morbidity, reductions between 30% and 60% have been reported.^12^,^13^,^14^ In addition, much higher reductions in the episodes of severe malaria were observed in separate studies by Diawara et al (2019) and Cisse et al (2016) compared to what was observed in this study (62% and 45% versus 10%). The observed mortality reduction was similar to the findings of Issiaka et al (2020) [67% versus 66%].^15^

The finding variations observed by different studies may lie in the design and the level of implementation and acceptability of other malaria control interventions prior to the introduction of SMC. As stated earlier, the malaria control interventions are complementary and in most sub-Saharan African countries mass distribution of long-lasting insecticide-treated nets (ITNs) is a basic public health intervention against the disease.^16^,^17^ Its use ranges between 3%-80%.^16^ Acceptability and appropriate use often reflect disease burden and determine the extent to which additional tools impact control: where the parameters are low, the disease burden is likely to be high and vice versa.^17^

SMC campaigns are benchmarked against set targets to assess programme performance at the regional, district and sub-district: at least 90% of eligible children must be dosed per cycle and the average number of SPAQ dose packs received per child should not be less than three for the entire (four cycles) campaign.^10^ Although eligible children received an average of 3.7 dose packs, the regional coverage for the first cycle was less than 90%. The low coverage was attributed to caregiver hesitancy due to the novelty of intervention and speculations that it was inimical to the health of children.

With follow-ups and advocacy, some refusals were over-turned but others remained adamant until subsequent cycles; having been convinced by the ‘testimonies' of care-givers who accepted the treatment. . The general drop in coverage for the fourth cycle was mainly due to volunteer exhaustion and overwhelming access challenges at the inception of the floods and overflow of rivers during the peak of the rainy season.

Three districts (Bole, Central Gonja and East Gonja) consistently achieved perfect coverage and even crossed the 100% mark for some of the cycles. The observation raises flag on adherence to SMC standard operation procedures and reliability of the population projections assigned to districts and regions. Dosing ineligible children (outside the target age); multiple data entries; application of underestimated population targets; and influx from adjoining communities may underly the observation. However, because the SMC campaign periods overlap in the five northern regions the latter may not be a significant contributor.

The need to address gaps in the estimation of the target population and improve service data management would facilitate monitoring of programme performance and impact assessment. The engagement of satisfied caregivers as advocates may be explored to reduce hesitancy and refusals as well as contribute to sustaining the acceptability of the intervention. Lastly, expansion of the intervention to other high-prevalence regions with seasonal variation in disease burden may be worthwhile.

A limitation of the study was that we are unable to ascertain whether children who developed malaria or died from it received SMC (and for how many months in the intervention year) due to the characteristics of the source data – aggregated data.

Again, we utilized under-five malaria morbidity and mortality data (instead of 3–59 months) due to data separation challenges. This might not affect validity of the study because of low incidence of malaria and related mortality among children under three months.^18^

## Conclusion

Four cycles of SMC reduced the under-five malaria morbidity and mortality in the Savannah Region by 17% and 67% respectively during the first year of implementation. The intervention (combined with existing control measures) wields prospects of accelerating the regional/national malaria elimination efforts if the implementation is optimised.
